# Targeted Separation of COX-2 Inhibitor from *Pterocephalus hookeri* Using Preparative High-Performance Liquid Chromatography Directed by the Affinity Solid-Phase Extraction HPLC System

**DOI:** 10.3390/molecules26237395

**Published:** 2021-12-06

**Authors:** Yunhe Zhu, Weidong Wang, Lei Jiang, Hui Tan, Zenggen Liu, Sirong Jiang, Yanduo Tao, Huaixiu Wen, Lijuan Mei

**Affiliations:** 1 Key Laboratory of Tibetan Medicine Research, Northwest Institute of Plateau Biology, CAS, Xining 810001, China; zhuyunhe9806@163.com (Y.Z.); wangweidong@nwipb.cas.cn (W.W.); zhuyunhe@nwipb.cas.cn (L.J.); tanhui@nwipb.cas.cn (H.T.); lzg@nwipb.cas.cn (Z.L.); jiangsirong@nwipb.cas.cn (S.J.); tyd@nwipb.cas.cn (Y.T.); 2 Qinghai Provincial Key Laboratory of Tibetan Medicine Research, Xining 810001, China; 3 University of Chinese Academy of Sciences, Beijing 100049, China

**Keywords:** affinity screening, cyclooxygenase-2 inhibitors, preparative separation, *Pterocephalus hookeri*

## Abstract

*Pterocephalus hookeri*, as a kind of popular traditional Tibetan medicine, is reputed to treat inflammatory related diseases. In the present work, a cyclooxygenase-2 functionalized affinity solid-phase extraction HPLC system was developed and combined with preparative-HPLC for rapidly screening and separating cyclooxygenase-2 ligand from *P. hookeri* extracts. Firstly, ligands of cyclooxygenase-2 were screened from extracts by affinity solid-phase extraction HPLC system. Then directed by the screening results, the recognized potential active compounds were targeted separated. As a result, the major cyclooxygenase-2 inhibitor of *P. hookeri* was obtained with a purity of >95%, which was identified as sylvestroside I. To test the accuracy of this method, the anti-inflammatory activity of sylvestroside I was inspected in lipopolysaccharide-induced RAW 264.7 cells. The results show that sylvestroside I significantly suppressed the release of prostaglandin E_2_ with dose-dependent, which was in good agreement with the screening result of the affinity solid-phase method. This method of integration of screening and targeted separation proved to be very efficient for the recognition and isolation of cyclooxygenase-2 inhibitors from natural products.

## 1. Introduction

Traditional Tibetan medicines (TTMs) are valuable for the treatment of plenty of clinical diseases in China [1]. *Pterocephalus hookeri* (*P. hookeri*) is a kind of popular TTM for treating inflammation-related diseases, such as rheumatoid arthritis, influenza, and enteritis [2,3,4]. Additionally, modern pharmacological research has proven that *P. hookeri* possesses anti-inflammatory [5], analgesic [6], anti-tumor [7,8], antimicrobial [9], antiangiogenic [2] and neuroprotective activities [10]. At present, most of the compounds isolated from *P. hookeri* are iridoids [11], such as sweroside [3], pterocenoids A [12], and hookerinoids A [13]. At the same time, studies have shown that iridoids were the main material basis for *P. hookeri* to exert the above medicinal effects [14]. In addition, it also includes triterpenes [4], phenylpropanoids [3], flavonoids [15], etc. However, a vast amountof research on the activities of *P. hookeri* are based on the crude extracts, and only a few studies have analyzed the activity of individual compounds [5,16]. It is desirable to find out the specific chemical components from *P. hookeri* related to the activities.

Thousands of enzymes with diverse structures and different functions play a vital role in metabolism, but the expression or function of some enzymes would be abnormally increased/decreased in disease [17]. At present, at least 1500 kinds of enzymes have been identified as targets for disease prevention and treatment [18]. The cyclooxygenase (COX) enzymes catalyze arachidonic acid (AA) into prostaglandins (PGs) affects virtually all known physiological and pathological processes as autocoid mediators [19]. Nonsteroidal anti-inflammatory drugs (NSAIDs) exert anti-inflammatory, analgesic and antipyretic action through inhibition of PGs production secondary to their inhibition of COX [20]. COX isoenzymes not only include the constitutive COX-1 but also the inducible COX-2, which is usually very low under physiological conditions and elevates during the inflammation progress [21]. The COX-2 enzymes are clinically important because the inhibition of COX-2 confers relief from inflammatory, pyretic, pain and oncological maladies [22]. However, due to COX-1 inhibition, traditional NSAIDs cause side effects such as gastrointestinal bleeding, ulcerations and renal damage [23]. Thus, there has been ever increasing interest in development of COX-2 inhibitors, and the study evaluates and screens the crude extract of *P. hookeri* with COX-2 as the target to develop the selective COX-2 inhibitors.

The general mode of plant-derived compound discovery with COX-2 inhibitory activity generally requires multiple-step separation and activity determination of every separated molecule, which is time consuming, labor-intensive and leads to low efficiency [24]. To enhance the discovery throughput of COX-2 inhibitors, plenty of methods are based on biological binding affinities between ligands and enzymes, for directly screening the potential COX-2 inhibitors from a complex mixture are developed. Among them, the affinity solid-phase extraction HPLC (ASPE-HPLC) system has been extensively used due to its online detection and good reproducibility [25,26,27]. ASPE technology is designed to immobilize the drug target (enzyme) on the surface of solid-phase extraction column packing, and discriminate between complex systems based on the affinity between enzyme and small molecules [26]. The ASPE column is installed in a high-performance liquid chromatograph for real-time observation and collection of elution. Then, the next step is to identify the ligands by HPLC analysis. At present, potential ligands of α-glucosidase [26], xanthine oxidase [25], and acetylcholinesterase [27] have successfully screened through the affinity solid-phase extraction HPLC system. However, this affinity solid-phase extraction HPLC system is suitable for sample screening and bioassay. Other technologies are needed to separate the potential COX-2 inhibitors screened by this system for subsequent experiments.

Preparative HPLC (pre-HPLC), as a separation technique, is widely employed for purifying compounds with a high purity from the crude extraction of TTMs [28,29]. This method is detected online with advantages of superior performance, excellent reproducibility and automation [30]. Furthermore, when the elution of the sample is enlarged from the analysis stage to the preparative separation stage, the chromatograms could be basically consistent as long as they are without alteration of the composition of mobile phase [31]. However, it is difficult to obtain the target compounds with high purity only by preparation when natural products are complex. In general, through enrichment before the purity preparation, the target compounds could be enriched and non-target ingredients are removed. Then, preparation can substantially purify the target compound with a purity of >95% [32]. The medium-pressure chromatographic tower is based on the absorption chromatography with the advantage of higher sample loading, the convenience of packing procedure, lower cost, and online detection and has widely used for crude extracts purification and natural compounds enrichment [15,28,33]. Therefore, it is necessary to isolate the potential COX-2 inhibitors by pretreatment of medium-pressure chromatographic tower and suitable preparative HPLC strategy for chemical structural characterization and inhibitory activity verification.

In this study, in order to screen for active compounds with COX-2 inhibitory activity from *P. hookeri* and further explain its traditional use, a COX-2 functionalized the affinity solid-phase extraction HPLC system was developed to screen and characterize the cyclooxygenase-2 ligand from *P. hookeri* ethanol extracts pretreated via medium-pressure chromatographic tower. Potential COX-2 inhibitors were screened and separated rapidly using the ASPE-HPLC system coupled with a preparative HPLC technique. Furthermore, the structure and the in vitro COX-2 inhibitory activity of the active compound are determined. This advanced method, for the target separation of COX-2 inhibitors from complex extracts, could be applied for the isolation of ligands from natural productors in a highly efficient manner.

## 2. Materials and Methods

### 2.1. Instrumentation and Reagents

HPLC analysis was implemented by a Shimadzu series SIL-16 (Shimadzu, Kyoto, Japan). The enrichment and preparation were implemented by a Hanbon series NP7005C (Hanbon Sci & Tech, Huai’an, China). The NMR spectra were recorded on deuterated DMSO-*d*_6_ by Bruker Avance 600 MHz instrument (Ammerbuch, Germany). ESI-MS spectra were recorded on an Agilent 6546 LC/Q-TOF (Santa Clara, CA, USA). Odyssil C18 (4.6 × 250 mm, 5 μm) analysis column, Odyssil C18 (20 mm × 250 mm, 5 μm) preparative column and Phecda C18 (20 mm × 250 mm, 10 μm) preparative column were purchased from Agilent Technologies (Santa Clara, CA, USA). Silica gel (5 μm, 300 Å), AB-8 macroporous resin, and SBC MCI gel inverse packing were bought from Agricultural Chemical Co. Ltd. (Tianjin, China). Sodium dihydrogen phosphate and hydrogen phosphate were bought from Sinopharm Chemical Reagent Co., Ltd. (Shanghai, China). Preparative ACN was purchased from Tianjin Factory (Tianjin, China). Deionized water (18.5 MΩ) was purified using PAT-125 machine fabricated by Chengdu Ulupure Technology (Chengdu, China).

The RAW 264.7 cell line was obtained from the Chinese Academy of Science Cell Bank (Shanghai, China). Mouse anti-rabbit COX-2 primary antibody (#12282), β-actin antibody and secondary antibodies were obtained from Inc. (Boston, MA, USA). The mouse prostaglandin E_2_ (PGE_2_) ELISA Kit assay kit, COX-2 inhibitor screening kits and the reagents used to culture cells are from Beyotime (Shanghai, China).

### 2.2. Sample Extraction and Pretreatment

*P. hookeri* herbs were collected from Batang Township, Yushu County, Qinghai Province (longitude: 96.9809, latitude: 32.8599, altitude: 3900 m) on 18 August 2020 and were validated by Professor Lijuan Mei (Northwest Institute of Plateau Biology, Chinese Academy of Sciences). The specimen (No.0358208) was stored in the Qinghai-Tibetan Plateau Museum of Biology.

Note that 500 g of air-dried and pulverized *P. hookeri* were mixed with 4 L of 95% ethanol (the ratio of 1:8) for 24 h three times. The total solutions were rotary evaporated at 55 °C in vacuum. The sample resuspended in deionized water was loaded in a microporous resin column (10 cm × 50 cm, 4 L) and eluted with ethanol/water mixture (0:100 and 95:5 *v*/*v*). Then, samples concentrated from the 95% ethanol/water eluentions were dissolved in methanol and filtered through a 0.45 μm filter for further experiments.

### 2.3. Preparation of Affinity Solid-Phase Extraction Column

Silica gel (200 mg) was mixed in 150 mL of ethanol for 10 min. Then added APTES (4.00 mL) under 700 r/min mechanical stirring and reacted for 6 h. The silica gel was filtered and washed with 200 mL of ethanol and water, respectively, and dried in an oven at 50 °C. The amino-functionalized silica gel was dispersed in 150 mL of 5% glutaraldehyde solution for 1 h at room temperature. After incubation, the aldehyde-based silica gel was washed again, then dispersed in 150 mL of 12.6 U/mL COX-2 solution and shaken for 2.5 h. The COX-2 modified silica gel was filtered and washed, and the unfunctionalized aldehyde group was blocked with 150 mL of 0.2 mol/L ethanolamine solution for 6 h. Finally, it was packed into a stainless-steel column (4.6 mm × 30 mm) by the wet packing method to obtain an affinity solid-phase extraction column. The effectiveness of the COX-2 affinity solid-phase extraction column was investigated by a positive control (celecoxib) and a negative control (glipizide) of the COX-2 inhibitor. Additionally, the specific experiments and results were in the Supplementary Materials.

### 2.4. Chromatographic Conditions

The crude sample solution was loaded onto a preprocessed MCI column (49 mm × 460 mm) and eluted in ethanol/water. A linear gradient elution was performed for 0–30 min (0–40% ethanol), 30–130 min (40–55% ethanol) and 130–140 min (55–100% ethanol) at a flow rate of 30 mL/min.

The screening of the extract Fr-3 is carried out on the affinity solid-phase extraction HPLC system. The determination of the COX-2 inhibitory activity was performed using the ASPE column. The mobile phases were PBS and methanol, and the gradient elution step was 100% PBS for 25.00 min, 100–0% PBS from 25.00 to 27.00 min, and 100% methanol from 27.00 to 50.00 min. The flow rate was 1 mL/min, and the chromatogram was recorded at 254 nm. Then HPLC analysis of the sample was performed on the Odyssil C18 column. The liquid chromatography conditions were as follows: the mobile phase consisted of 0.2% aqueous formic acid and ACN with a flow rate of 1 mL/min and the gradient elution: 0–50.00 min, 5–35% ACN. Chromatographic data were collected at 254 nm.

Separation of Fr-3 was implemented on the Odyssil C18 preparative column using a mobile phase composed of 0.2% aqueous formic acid and ACN, and the gradient elution step was 5–35% ACN for 50.00 min. The flow rate was 19 mL/min. The target fraction (Fr-t) was obtained through this enrichment preparation. Then the isolation of Fr-t was performed on the Phecda C18 preparative column. The liquid chromatography conditions were as follows: the mobile phases were 0.2% aqueous formic acid and ACN, with a flow rate of 19 mL/min and the isocratic elution was 23% ACN for 50.00 min. Chromatograms were collected at 254 nm.

MS data were acquired across the range *m/z* 100–1000 in negative ion modes with an acquisition rate of 1.03 spectra/s. The operating conditions were as follows: the pressure of the nebulizer, 35 psi; capillary voltage, 4000 V; skimmer, 60 V; and fragment voltage, 135 V. The ions were analyzed using the TOF analyzer. Additionally, the mass axis was calibrated using a mixture provided by the manufacturer.

### 2.5. Determination of Anti-Inflammatory Activity

#### 2.5.1. COX-2 Inhibitory Activities Assay

The Fr-3 were dissolved in DMSO at the concentration of 2.0 mg/mL as a stock solution and the COX-2 inhibitory activity of various final concentrations (0.63, 1.25, 2.5, 5.0, 10 μg/mL) were determined by using COX-2 inhibitor screening kits. The activity of COX-2 inhibitors could be detected very sensitively by fluorescence detection (Ex 560/Em 590).

#### 2.5.2. Cell Culture and Treatment

Sylvestroside I was dissolved in water at a concentration of 120 mM as a stock solution. RAW 264.7 cells were cultured in DMEM supplemented with 10% fetal bovine serum, 100 U/mL penicillin and streptomycin. Cells were incubated at 37 °C with 5% CO_2_.

#### 2.5.3. Cell Viability Assay

MTT assay was used to detect the cell viability and evaluate the cytotoxicity. RAW 264.7 cells were treated with sylvestroside I at final concentrations of 0.0, 0.45, 0.9, 1.8, 3.6 mM. Following 24 h of incubation, MTT solution was added at a final concentration of 50 μg/mL and the cells were incubated for another 4 h. Then, 100 μL of DMSO was added to dissolve the formazan. The cell viability could be detected by a microplate reader at a wavelength of 570 nm. The untreated cells were considered to be 100% viable cells, and results are expressed as the percentage of viable cells compared with untreated cells.

#### 2.5.4. PGE_2_ Levels Analysis

RAW 264.7 cells were treated by lipopolysaccharide (LPS) of 1 μg/mL with or without Sylvestroside I (final concentrations 0.45, 0.9, 1.8, 3.6 mM) for 24 h. The normal control cells were set in the control group. PGE_2_ levels were determined using the PGE_2_ ELISA kit with the multifunctional microplate reader.

#### 2.5.5. COX-2 Levels Analysis

The RAW 264.7 cells with sylvestroside I (final concentrations 0.45, 0.9, 1.8, 3.6 mM) treatments for 24 h were lysed in a RIPA lysis buffer to produce the proteins. The equal amounts of protein were boiled with SDS-PAGE loading buffer. Then all sample proteins were separated by electrophoresis on 10% SDS-PAGE and transferred into PVDF membranes. The membranes were blocked for 2 h with 10% BSA-TBST. After blocking, the membranes were incubated with the COX-2 and β-actin antibodies overnight. Then, peroxidase-conjugated mouse secondary antibodies were incubated with the membranes for 4 h. The results were detected by enhanced chemiluminescence reagent and exposed to an imaging system.

#### 2.5.6. Molecular Docking

The crystal structure of COX-2 was obtained from the PDB database (PDB ID: 5KIR, https://www.rcsb.org/structure/5KIR, latest accessed on 20 November 2021). And the compound Sylvestroside I for molecular docking was downloaded from the PubChem database (https://pubchem.ncbi.nlm.nih.gov/compound/101967019, latest accessed on 20 November 2021).

Molecular docking was carried out using AutoDock Vina 1.1.2. Before docking, the protein was prepared using the PyMOL 2.5 software to remove water molecules and other undesirable structures. Then, the binding pocket was defined by the crystal ligand, and the box enclosing the pocket was set at a size of 30 × 30 × 30 Å. Therefore, small molecules and proteins files were converted to the PDBQT format by the ADFR suite 1.0 software^3^. Finally, docking was utilized to conduct semi-flexible docking with a maximum of 50 poses output after internal clustering. The best scoring conformation was further visualized based on PyMOL 2.5.

## 3. Results and Discussion

### 3.1. P. hookeri Extract Pretreatment by Medium-Pressure Chromatography

**Figure 1:**
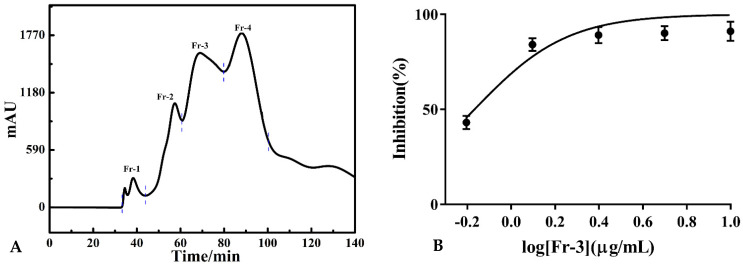
Pretreatment chromatogram of ethanol extract of *P. hookeri* with MCI medium-pressure chromatographic tower (**A**); The dose-response curves and IC_50_ value of Fr-3 (**B**).

*P. hookeri* is rich in anti-inflammatory components, which were extracted by ethanol and enriched by microporous resin. However, there were still some impurities (biomacromolecules, fat-soluble components, etc.) in the ethanol crude extract of *P. hookeri* that would affect the separation effects and lead to column contamination. Thus, the online MCI medium-pressure chromatographic tower was used to purify crude extracts before preparative HPLC. This online medium-pressure column could enhance the separation efficiency by achieving visual separation and removing nontarget components of the sample. A total of 10.00 mL of the sample (0.40 g/mL) was added to the MCI medium-pressure chromatographic tower and eluted with ethanol and water. The elution condition is shown in Section 2.4 and the separation chromatogram is shown in Figure 1A. After 15 repeated injections, 4 fractions were obtained. A total of 10.19 g of fraction-3 (Fr-3) was collected and the recovery rate was approximately 16.98%. Fr-3 of *P. hookeri* extract showed strong inhibition on COX-2 with IC_50_ value of 0.68 μg/mL, which encouraged us to look for COX-2 inhibitors from it. Figure 1B showed the inhibition curve and IC_50_ value of Fr-3. Then, Fr-3 was dissolved in methanol and strained through 0.45 μm filters for further screening, which remains unexplored to date.

### 3.2. Identification of COX-2 Inhibitors with the ASPE-HPLC System

**Figure 2:**
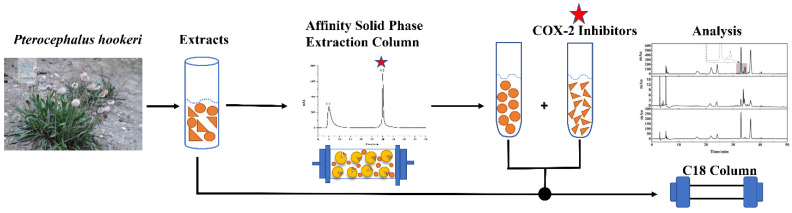
Schematic illustration of the affinity solid-phase extraction HPLC system for screening COX-2 inhibitors from Fr-3 of *P. hookeri.* The orange shapes represent compounds in Fr-3, and the yellow shapes represent COX-2 (human, recombinant).

**Figure 3:**
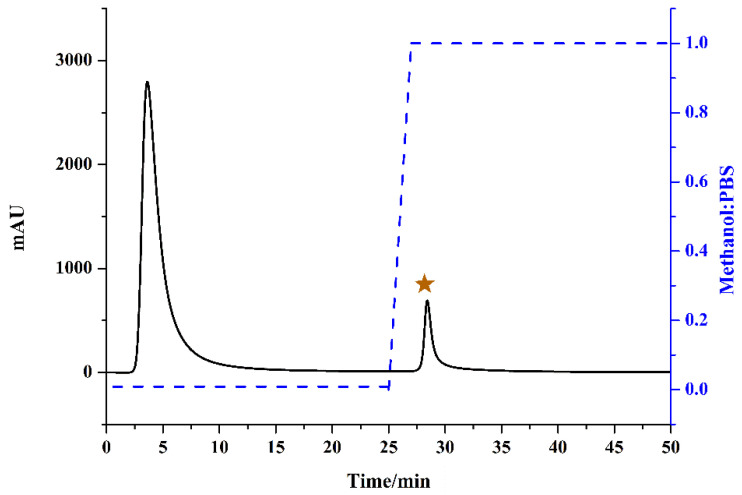
Chromatogram (black line) and concentration ratio of methanol and PBS (blue line) on the affinity solid-phase extraction column for Fr-3 of *P. hookeri.* The chromatographic conditions: eluent A: PBS, B: methanol; gradient: 0–25 min, 100% A; 25–27 min, 100–0% A; 27–50 min, 100% B; flow rate: 1 mL/min; monitoring wavelength: 254 nm.

The affinity solid-phase extraction HPLC method for screening and analyzing COX-2 inhibitors is depicted in Figure 2. The ASPE column packed with silica gel which functionalized with COX-2 by the glutaraldehyde cross-linking method is critical to this strategy [15]. Experimental details of the ASPE column fabrication referenced from the literature with slight modifications [25]. When fraction-3 of *P. hookeri* was loaded in the ASPE column, ligands bound to the enzymes and unbound compounds were eluted by PBS buffer. As shown in Figure 3, unbound small molecules mainly eluted in 2.60–6.20 min and almost all compounds were eluted except the ligands which remained on the affinity column at 25.00 min. Then ligands needed to be dissociated and enriched. Methanol is proven as a good solvent for quickly dissociate bound ligands [25,34]. Thus, methanol was chosen for elution after 25.00 min, and the ligands were mainly eluted from 27.80 to 29.30 min (Figure 3). Although COX-2 protein tends to denaturize in the organic solvent environment, the chemical properties of polypeptide chains did not change (no inactivation). If the factors causing deformation are removed and a suitable folding environment (such as PBS buffer) is established, the unfolded proteins could spontaneously refold and restore their natural state [35]. The immobilized COX-2 remained at great repeatability after 15 injections (result not shown), which indicated that COX-2 functionalized silica gel was repeatable and cost-effective.

**Figure 4:**
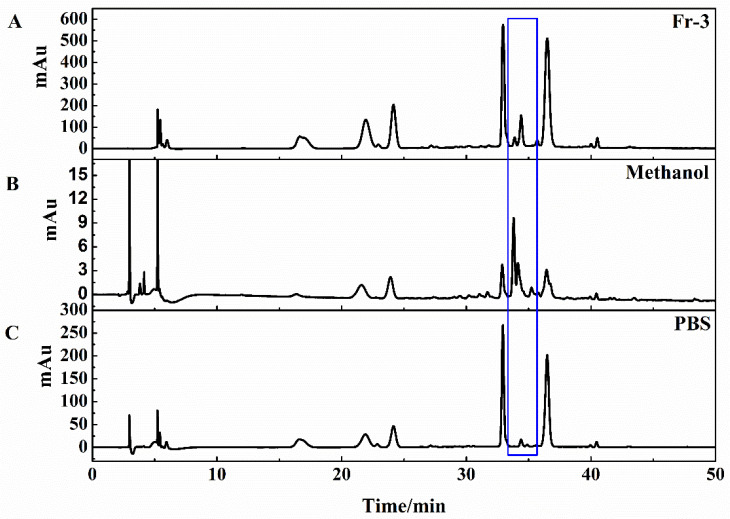
The HPLC analysis chromatogram of *P.hookeri* extract Fr-3 (**A**), methanol elution component (**B**) and PBS elution component (**C**) on the Odyssil C18 analytical column. Condition: mobile phase A: 0.2% *v*/*v* formic acid/water, B: ACN; gradient: 0–50 min, 5–35% B; monitoring wavelength: 254 nm; flow rate: 1.00 mL/min. The target fraction was framed by the blue line.

Generally, peak area ratios of components which exhibited higher binding affinity capacities with target enzymes would be significantly different from that of lower or no binding abilities [36]. The HPLC chromatogram of Fr-3, methanol elution component and PBS elution component are shown in Figure 4. Figure 4B shows that the relative areas of target fraction were increased obviously compared with the HPLC chromatogram of *P. hookeri* extract. At the same time, the relative areas of the target fraction were obviously reduced in the HPLC profile of PBS elution component (Figure 4C). This result revealed that the target fraction originating from COX-2 inhibitors were recognized from the extract of *P. hookeri.*

### 3.3. Enrichment of COX-2 Inhibitors Fraction

**Figure 5:**
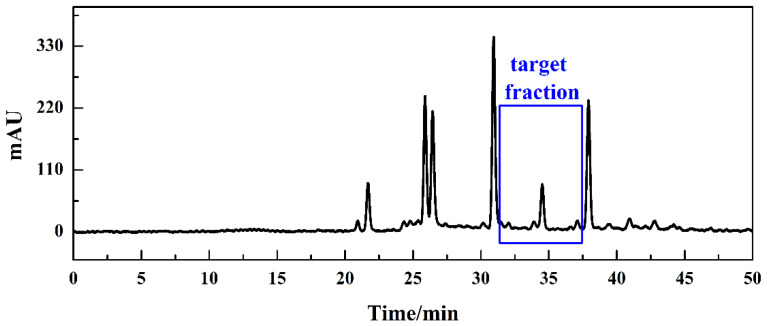
The separation chromatogram of sample Fr-3 on the Odyssil C18 preparative column. Condition: mobile phase A: 0.2% *v*/*v* formic acid/water, B: ACN; gradient: 0–50 min, 5–35% B; monitoring wavelength: 254 nm; flow rate: 19.00 mL/min. The target-fraction was framed by the blue line.

After successfully screening of the active compound by the affinity solid-phase extraction HPLC system, a reliable means of achieving high-purity isolation of specific compounds was needed. Additionally, because the COX-2 inhibitory activity of Fr-3 mainly originated from the fraction which is not the main component of the *P. hookeri* extract between two main peaks, it is important to enrich the target fraction. Preparative HPLC is suitable for the precise isolation and detailed purification of trace compounds due to high recovery rate, real-time detection and excellent reproducibility. Therefore, we combined the ASPE-HPLC system with preparative-HPLC to efficiently separate potential COX-2 inhibitors from *P. hookeri*. As shown in Figure 5, the preparative Odyssil C18 column chromatogram was basically consistent with the analytical chromatogram in the retention time. To prevent the potential COX-2 inhibitors missing, target-fraction (Fr-t) with elution time ranging from 31.40–37.50 min were collected. Following preparation, the solution was concentrated under decompression and a total of 1.32 g of the Fr-t was obtained.

### 3.4. Purification and Characterization of COX-2 Inhibitor

**Figure 6:**
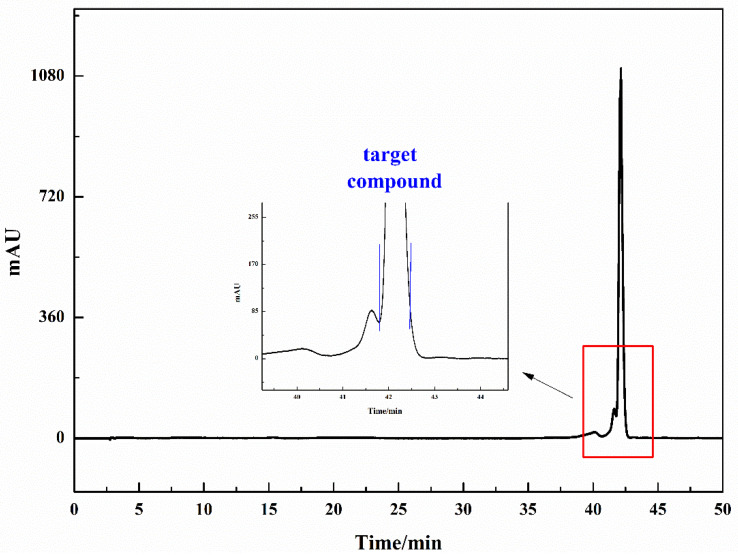
The separation chromatogram of sample Fr-t on the Phecda C18 preparative column. Condition: mobile phase A: 0.2% *v*/*v* formic acid/water, B: ACN; isocratic elution condition: 0–50 min, 23% B; monitoring wavelength: 254 nm; flow rate: 19.00 mL/min. The target compound was framed by the blue line.

The purpose of the enrichment step was to concentrate the target compounds and make them become the main compound in this fraction. The next step was to prepare the active compound with high purity. In order to efficiently purify the target compound, Fr-t was prepared using the Phecda C18 column, and the preparative chromatogram is shown in Figure 6. This RPLC/RPLC approach is suited to separating nonpolar, polar and ionic compounds from natural products. Additionally, when the sample contained less than ten weakly retained compounds, isocratic separation had a higher separation degree and feasibility compared to gradient separation [37]. The gradient experiment result could be used to predict the isocratic retention of certain compound at a constant mobile phase composition. In order to efficiently purify the target compound, isometric elution condition (0–50.00 min, 23% ACN) was used, and the mobile phase was composed of 0.2% *v*/*v* formic acid/water and ACN.

**Figure 7:**
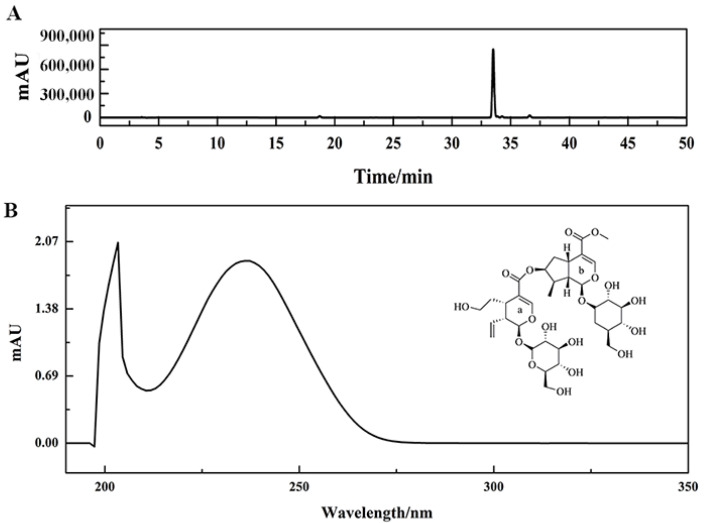
The purity analysis of isolated potential COX-2 inhibitor using the Odyssil C18 analysis column (**A**). Condition: mobile phase A: 0.2% *v*/*v* formic acid/water, B: ACN; gradient: 0–50 min, 5–35% B; monitoring wavelength: 254 nm; flow rate: 1.00 mL/min. The ultraviolet-visible absorption spectrum and chemical structure of potential COX-2 inhibitor (**B**).

Finally, 765.66 mg of active compound with a purity higher than 95% was isolated. Its purity test diagram and ultraviolet-visible spectroscopy are shown in Figure 7A,B, respectively. This potential COX-2 inhibitor was determined as sylvestroside I by comparing NMR spectral data with published literature.

Target compound (sylvestroside I, C_33_H_48_O_19_, ESI-MS *m*/*z* 747.2717 [M-H]^-^, transparent sticky block): ^1^H-NMR (600 MHz, DMSO-*d*_6_, *δ*, ppm, *J*/Hz): 0.98 (3H, d, *J* = 6.6, H-10b), 1.56 (1H, m, H-6a2), 1.71 (1H, m, H-6b2,) 1.97 (1H, m, H-6a1), 2.03 (1H, m, H-9b), 2.17 (1H, m, H-8b), 2.55 (1H, m, H-6b1), 2.68 (1H, m, H-9a), 2.97 (1H, s, H-5a), 2.99 (1H, m, H-5b), 3.16–3.37 (8H, Glc protons), 3.43 (4H, m, H-6′a, H-6′b), 3.63 (3H, s, H-12b), 3.69 (2H, m, H-7a1, H-7a2), 4.50 (1H, d, *J* = 7.9, H-1′a), 4.54 (1H, d, *J* = 7.9, H-1′b), 5.10 (1H, s, H-7b), 5.21 (2H, m, H-10a1, H-10a2), 5.29 (1H, m, H-1b), 5.46 (1H, d, *J* = 6.6, H-1a), 5.70 (1H, m, H-8a), 7.41 (1H, s, H-3b), 7.48 (1H, s, H-3a); ^13^C-NMR (MHz, DMSO-*d*_6_, *δ*, ppm): 96.34 (C-1a), 152.12 (C-3a), 110.81 (C-4a), 30.20 (C-5a), 33.51 (C-6a), 59.72 (C-7a), 135.36 (C-8a), 45.64 (C-9a), 119.10 (C-10a), 166.42 (C-11a), 99.20 (C-1′a), 73.57 (C-2′a), 77.17 (C-3′a), 70.55 (C-4′a), 77.76 (C-5′a), 61.65 (C-6′a), 96.04 (C-1b), 151.40 (C-3b), 111.69 (C-4b), 31.48 (C-5b), 39.15 (C-6b), 76.44 (C-7b), 43.78 (C-8b), 49.08 (C-9b), 13.82 (C-10b), 167.26 (C-11b), 51.51 (C-12b), 99.10 (C-1′b), 73.48 (C-2′b), 77.17 (C-3′b), 70.49 (C-4′b), 77.73 (C-5′b), 61.59 (C-6′b).

### 3.5. In Vitro COX-2 Inhibitory Activity Assay

**Figure 8:**
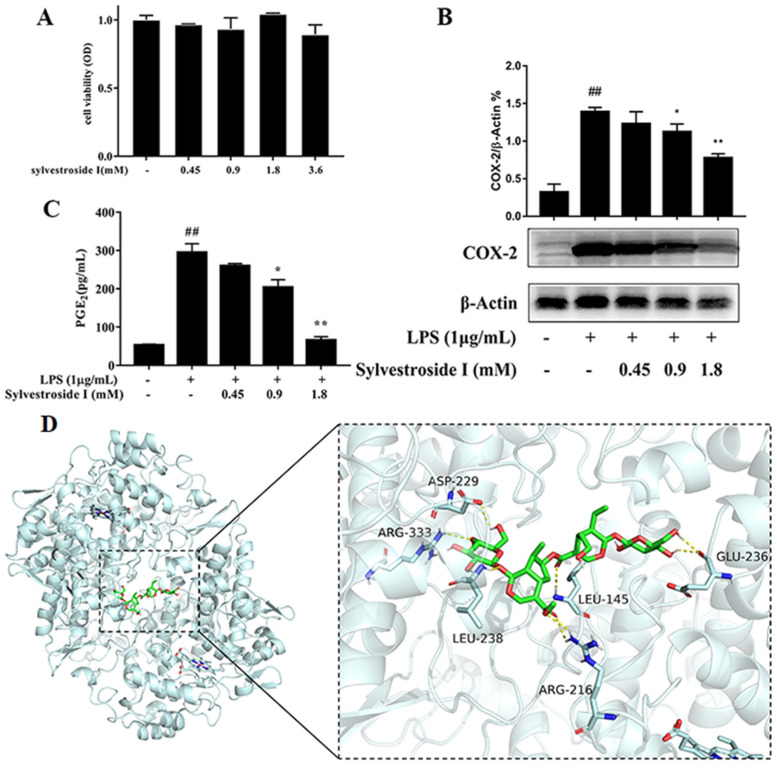
The cytotoxicity assay was conducted to measure the viability of RAW 264.7 cells with sylvestroside I (0.45–3.6 mM) for 24 h (**A**). The expression of COX-2 (**B**) and the PGE_2_ levels (**C**) was detected with the treatment of sylvestroside I (0.45–1.8 mM) in LPS-induced RAW 264.7 cells for 24 h. The results were expressed as mean ± SD (*n* = 3), ## *p* < 0.01 compared with the Con group; * *p* < 0.05, ** *p* < 0.01 compared with the LPS group. Docking model of sylvestroside I in COX-2 active site (**D**).

The conversions of AA to prostaglandin G_2_ and prostaglandin G_2_ to prostaglandin H_2_ were catalyzed by COX-2 enzyme [15]. In order to verify the screening result, sylvestroside I was tested its COX-2 inhibitory activity on the LPS-activated RAW 264.7 macrophage cells. As shown in Figure 8B, sylvestroside I could down-regulate the expression of COX-2 protein in a dose-dependent manner. Furthermore, the production of the inflammatory mediator PGE_2_, which was generated by the metabolism of AA through the COX-2, was downgraded by sylvestroside I in a dose-dependent manner (Figure 8C). Furthermore, the MTT results indicate that sylvestroside I has no obvious cytotoxic effect on the proliferation of RAW 264.7 cells at concentrations lower than 3.6 mM (Figure 8A), which showed that the suppression of COX-2 and PGE_2_ was not caused by the cytotoxicity of sylvestroside I [38]. Sylvestroside I was proved to possess anti-inflammatory activity which was in good agreement with the screening and targeted separation results. On the other hand, the molecular docking result showed that sylvestroside I was anchored at the COX-2 protein by multiple hydrogen bonds with ASP-229, ARG-333, LRU-145, LEU-238, ARG-216, and GLU-236 (Figure 8D). The binding energy is −8.8 kcal/mol, which indicated the binding of sylvestroside I with COX-2 is spontaneous.

In addition, the content of sylvestroside I in the whole grass was determined as 0.84 mg/g through HPLC, which indicated that sylvestroside I was one of the main anti-inflammatory constituents of *P. hookeri*. Therefore, via the ASPE-HPLC screening system combined with preparative HPLC, sylvestroside I, one of the main anti-inflammatory components in *P. hookeri*, was targeted isolated. Finally, the COX-2 inhibitory activity of sylvestroside I was confirmed in LPS-induced RAW 264.7 cells.

## 4. Concluding Remarks

In this study, we combined the COX-2 functionalized affinity solid-phase extraction HPLC strategy and preparative HPLC to screen and separate the potential COX-2 inhibitors from *P. hooleri* extract. Firstly, the sample of *P. hooleri* pretreated by medium-pressure chromatography was screened and recognized by the ASPE-HPLC system. Then the active compound was enriched and purified through preparative-HPLC and identified as sylvestroside I via the NMR spectral and MS spectra data. Finally, sylvestroside I without a cytotoxic effect was demonstrated to suppress COX-2 and PGE_2_ in LPS-induced RAW 264.7 cells. In summary, it was proved that the integrative strategy for the screen and subsequent separation of the potential COX-2 inhibitors from *P. hooleri* extract is a good guidance to search for anti-inflammatory compounds from TTMs of interest.

## Data Availability

The data and materials supporting the conclusions of this article are included within the article and Supporting Information.

## Supplementary Materials

The following are available online, Figure S1: The COX-2 affinity column chromatogram of mixture of celecoxib and glipizide (A); HPLC analysis of fractions C, C1, and C2. Figure S2: ^1^H NMR Spectrum (600 MHz) of Sylvestroside I (in DMSO-*d*_6_). Figure S3: ^13^C NMR Spectrum (600 MHz) of Sylvestroside I (in DMSO-*d*_6_). Figure S4: Negative MS spectra for Sylvestroside I. Figure S5: Original Western blots gel data of COX-2. Figure S6: Original Western blots gel data of β-actin.

## Sample Availability

Samples of the *P. hookeri*, COX-2 affinity solid-phase column and Sylvestroside I are available from the authors.

## Abbreviations

AA: arachidonic acid; APTES, 3-aminopropyltriethoxysilane; COX, cyclooxygenase; Fr-3, fraction-3; Fr-t, target-fraction; IC_50_, half-maximal inhibitory concentrations; LPS, lipopolysaccharide; PBS, phosphate buffered saline; PGs, prostaglandins; PGE_2_, prostaglandin E_2_; NSAIDs, nonsteroidal anti-inflammatory drugs; *P. hookeri*, *Pterocephalus hookeri;* The ASPE-HPLC system, the affinity solid-phase extraction HPLC system; TTM, traditional Tibetan medicine.
