# Supplementary material for: Targeted Separation of COX-2 Inhibitor from Pterocephalus hookeri Using Preparative High-Performance Liquid Chromatography Directed by the Affinity Solid-Phase Extraction HPLC System
Source: Molecules. 2021 Dec 6;26(23):7395. doi: 10.3390/molecules26237395 (PMC8658877; doi:10.3390/molecules26237395)

Supporting Information

Table of Contents

Figure S1 The COX-2 affinity column chromatogram of mixture of celecoxib and glipizide (A); HPLC analysis of fractions C, C1, and C2.

Figure S2 ^{1}H NMR Spectrum (600 MHz) of Sylvestroside I (in DMSO-*d6*)

Figure S3 ^{13}C NMR Spectrum (600 MHz) of Sylvestroside I (in DMSO-*d6*)

Figure S4 Negative MS spectra for Sylvestroside I

Figure S5 Original Western blots gel data of COX-2

Figure S6 Original Western blots gel data of β-actin

Experiments and results:

The mixture of celecoxib and glipizide (C) were loaded on the COX-2 affinity column and eluted by PBS and methanol. The gradient elution step was 100% PBS for 25.00 min, 100-0% PBS from 25.00 to 27.00 min, and 100% methanol from 27.00 to 50.00 min. the flow rate was 1 mL/min, and the chromatogram was recorded at 254 nm. As shown in Fig. S1A, two fractions C1 and C2 were obtained, and C2 were the active compounds that possess binding activity with COX-2. Then, fractions C, C1 and C2 were analysed by HPLC (chromatogram conditions: Odyssil C18 column; elution condition was 0-40 min, 5-95% ACN; flow rate was 1 mL/min; the chromatogram was recorded at 285nm). Results showed that the relative content of celecoxib in C2 is obviously improved compared in component C, which indicated that the celecoxib was retained on the COX-2 affinity column and needed elution with organic solvent (Fig. S1B). After screened by the affinity column, celecoxib and glipizide were distinguished successfully. This proved that it is effective to screen COX-2 inhibitors by the affinity column, and it also proved silica gel was successful modified of the COX-2 protein.

Figure S1 The COX-2 affinity column chromatogram of mixture of celecoxib and glipizide (A); HPLC analysis of fractions C, C1, and C2.

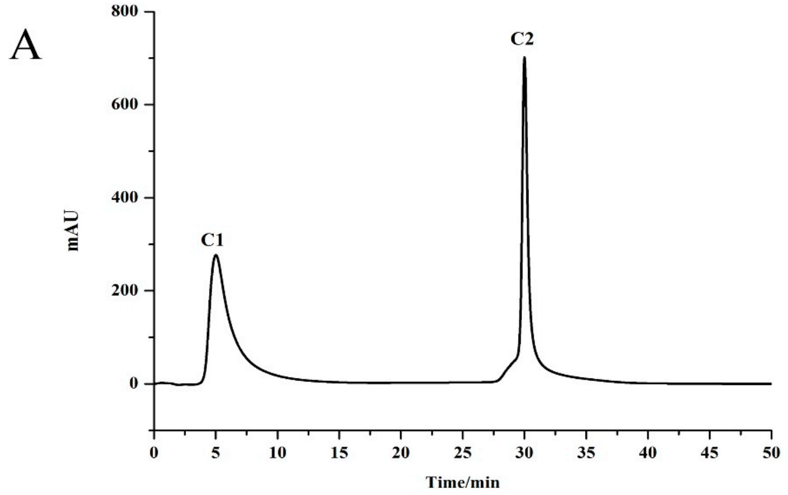

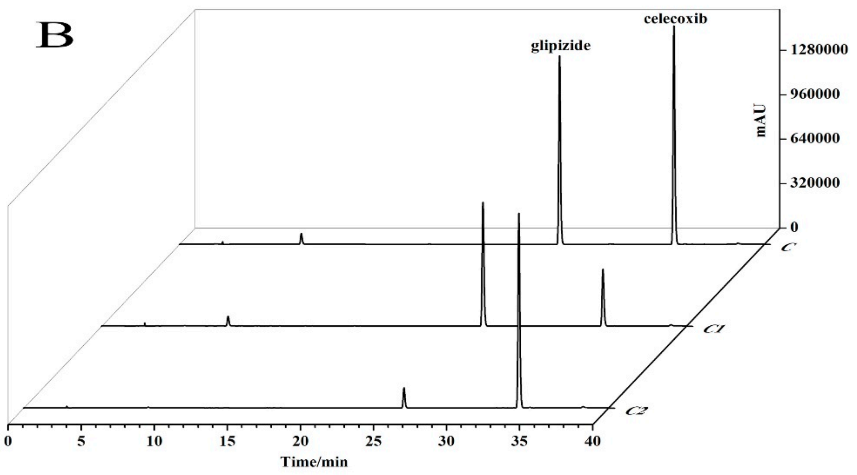

Figure S2 ^{1}H NMR Spectrum (600 MHz) of Sylvestroside I (in DMSO-*d6*)

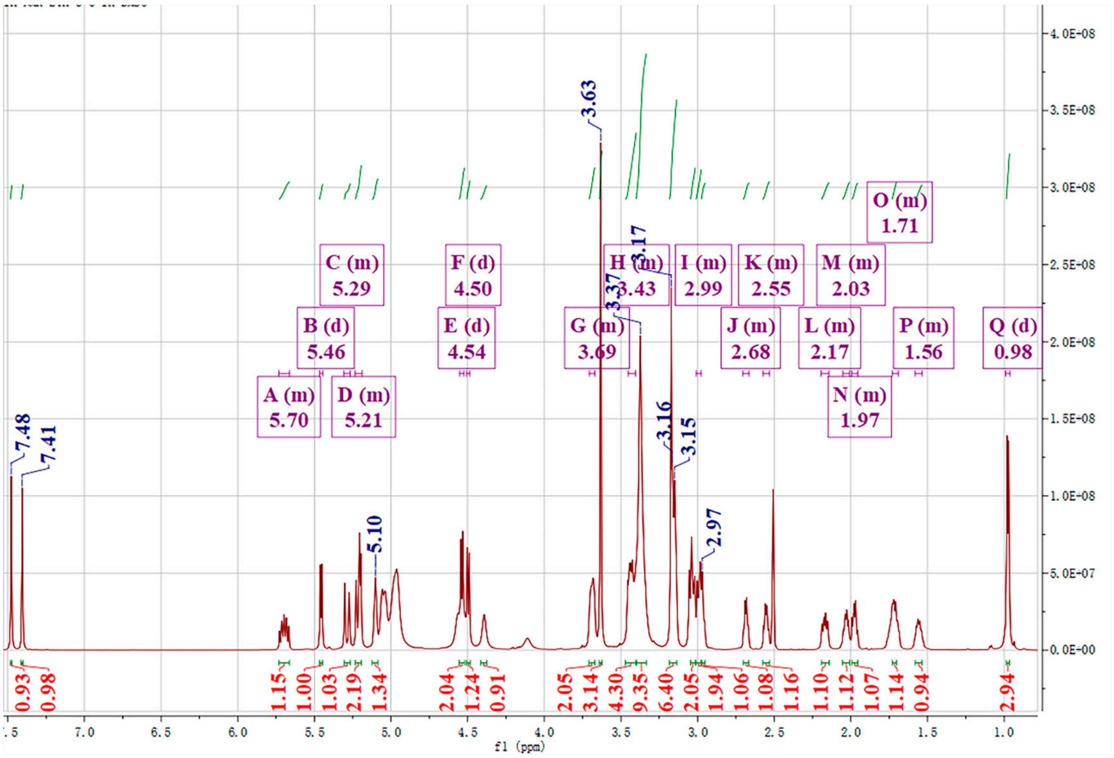

Figure S3 ^{13}C NMR Spectrum (600 MHz) of Sylvestroside I (in DMSO-*d6*)

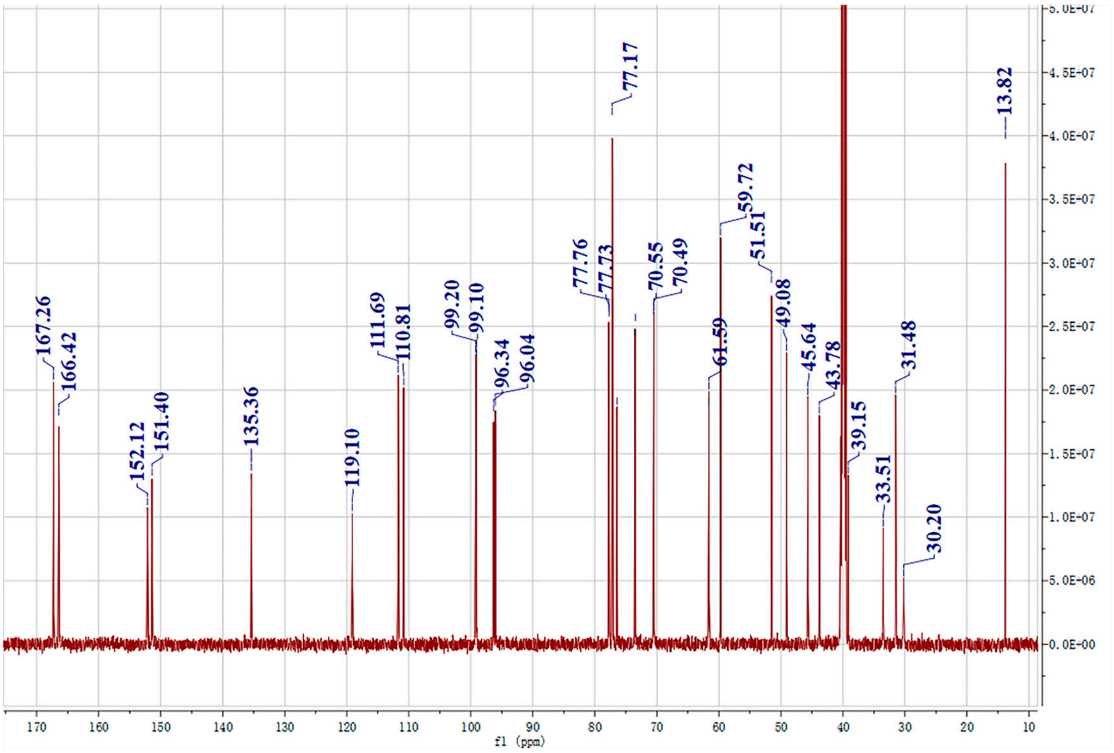

Figure S4 Negative MS spectra for Sylvestroside I

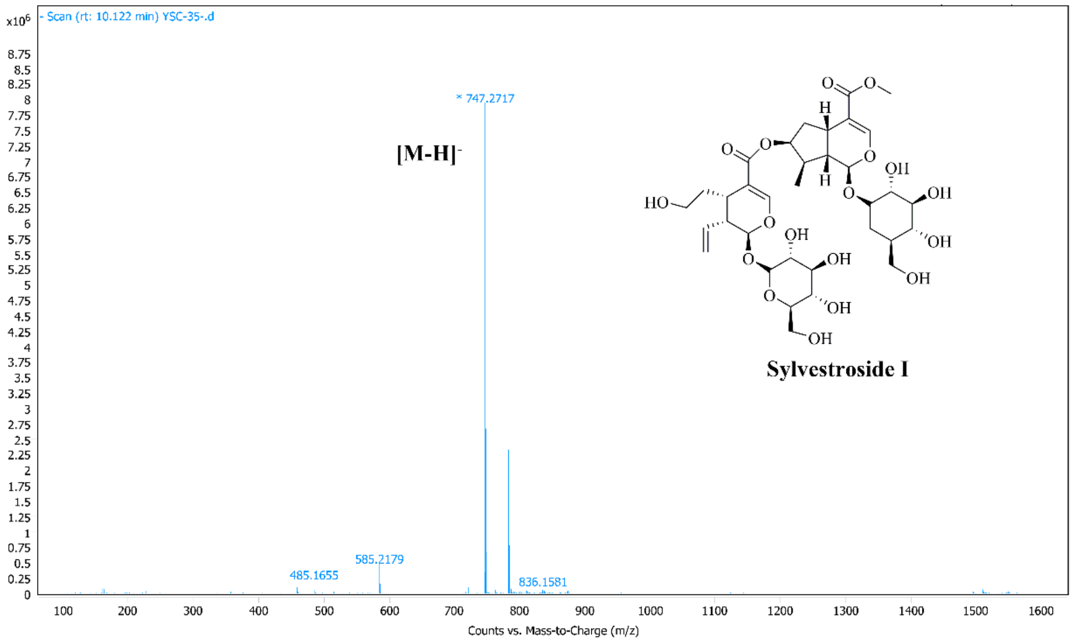

Figure S5 Original Western blots gel data of COX-2

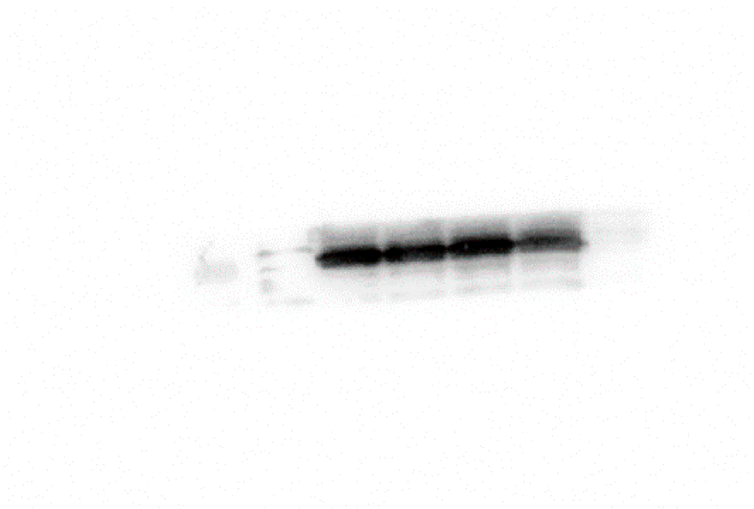

Figure S6 Original Western blots gel data of *β*-actin

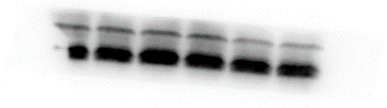

Supplement: Supplementary file 1 [file molecules-26-07395-s001.zip › molecules-1479952-supplementary.pdf]
